# Reply: Itkonen and Lamberg-Allardt’s Letter to the Editor Re: McClure et al. Nutrients 2017, 9, 95

**DOI:** 10.3390/nu9080804

**Published:** 2017-07-26

**Authors:** Scott T. McClure, Lawrence J. Appel

**Affiliations:** 1 Welch Center for Prevention, Epidemiology, and Clinical Research, Baltimore, MD 21205, USA; smcclur7@jhu.edu; 2 Department of Epidemiology, Johns Hopkins Bloomberg School of Public Health, Baltimore, MD 21205, USA; 3 Division of General Internal Medicine, Johns Hopkins University, Baltimore, MD 21205, USA

**Keywords:** phosphorus, bioavailability, dietary intake, nutrition databases, food ingredients

Dear Editor,

We appreciate Drs. Itkonen and Lamberg-Allardt’s interest in our recent article “Dietary Sources of Phosphorus among Adults in the United States: Results from NHANES 2001–2014” [1]. We agree with the importance of phosphorus bioavailability and added phosphates. However, we disagree with their conclusion that grain products are “not a particularly important source of bioavailable phosphorus” [2].

Total bioavailable phosphorus intake is the sum of bioavailable phosphorus from each source. See Equation (1) (calculation of bioavailable phosphorus intake from a specific dietary source), below. Note that total phosphorus intake from a specific source is the product of phosphorus content (mg/serving) and intake (servings/day).

Bioavailable Phosphorus Intake (mg/day) = Total Phosphorus Intake (mg/day) × Bioavailability (%)(1)

Identifying important dietary sources of total phosphorus intake is the first step in identifying important dietary sources of bioavailable phosphorus intake. Sources that contribute a small total amount of phosphorus (such as dark cola) necessarily contribute a small amount of bioavailable phosphorus, even if they have high bioavailability. Sources that contribute a large total amount of phosphorus (such as bread) may still contribute a large amount of bioavailable phosphorus with relatively low bioavailability. Our study highlights grain products as an increasingly important source of total dietary phosphorus intake.

Drs. Itkonen and Lamberg-Allardt also note the importance of added phosphates to the diet. Many foods, including grain products, contain a combination of different types of phosphorus (phytate and naturally-occurring or added phosphates). Added phosphates are of special interest due to the potential for food manufactures to increase or decrease their use more readily than naturally-occurring phosphates. The Food and Nutrient Database for Dietary Studies (FNDDS) does not currently provide the phosphorus content of foods specifically from added phosphates [3]. In the absence of values provided by food manufactures, a classification of each of the 8000+ food items in FNDDS is needed [3]. While we were not able to perform this classification in the current analysis, future research should attempt to classify types of phosphorus and estimate their intake.

Research into dietary intake of bioavailable and added phosphorus will benefit from an increased focus on important dietary sources of total phosphorus intake, such as grain products, rather than dietary sources with the highest phosphorus bioavailability.
